# Ambient air pollutants and hospital visits for pneumonia: a case-crossover study in Qingdao, China

**DOI:** 10.1186/s12889-020-10065-0

**Published:** 2021-01-07

**Authors:** Jianzhong Zhang, Dunqiang Ren, Xue Cao, Tao Wang, Xue Geng, Xin Li, Jinglong Tang, Shuguang Leng, Hongmei Wang, Yuxin Zheng

**Affiliations:** 1 Department of Occupational and Environmental Health, School of Public Health, Qingdao University, Qingdao, 266021 Shandong China; 2 Department of Respiratory Medicine and Critical care, The Affiliated Hospital of Qingdao University, Qingdao, 266003 Shandong China

**Keywords:** Air pollutants, PM_2.5_, PM_10_, Pneumonia, Case-crossover design, Principal component analysis

## Abstract

**Background:**

Pneumonia is one of the principal reasons for incidence and death in the world. The former research mainly concentrated on specific sources of patients. Besides, due to the heterogeneity among regions, there are inconsistencies in the outcome of these surveys. To explore the relationship between atmospheric pollution and hospital visits for pneumonia under the climate and pollution conditions in Qingdao, we carried out this study.

**Methods:**

The medical records of pneumonia patients were gathered from the affiliated hospital of Qingdao University during Jan 1st, 2014, and Dec 31st,2018. Daily concentrations of PM_2.5_, PM_10_, SO_2_, NO_2_, as well as CO, were collected from the national air quality monitoring stations in Qingdao. Case-crossover study design and conditional logistic regression model were used to estimate the associations. Daily temperature, relative humidity, and atmospheric pressure were adjusted as the covariates in all models. A principal component analysis was used to solve the multicollinearity between atmospheric pollutants and investigate the relationship between various air pollutants and pneumonia occurs.

**Results:**

In the single pollutant model, with interquartile range increment of the density of PM_2.5_, PM_10_, NO_2_ and SO_2_ at the lag2 days, the odds ratio of hospital visits for pneumonia patients increased by 6.4% (95%CI, 2.3–10.7%), 7.7% (95%CI, 3.2–12.4%), 6.7% (95%CI, 1.0–12.7%), and 7.2% (95%CI, 1.1–13.5%). Stratified analysis showed that pollutants were more significant in the cold period. Besides, the impact of atmospheric particulates on different ages mainly occurs in the young child (0 to 3-year-old). The odds ratio was 1.042 (95%CI, 1.012–1.072) when the principal components of atmospheric pollutants were included in the conditional logistic model.

**Conclusions:**

Our study found a significant relationship between short-term uncovering to PM_2.5_, PM_10_, NO_2_, SO_2_, and hospital visits for pneumonia in Qingdao. The effect of atmospheric pollutants mainly arose in a cold period. The particulate matter might be the principal reason in inducing hospital visits for pneumonia.

## Background

Pneumonia, a kind of inflammation of alveoli, terminal airway, and interstitial lung, is the principal cause for inducing onset and death in the world [1, 2]. Generally, about 2.5 million people suffering from pneumonia in China each year, and the number of deaths from pneumonia accounts for 17% of child deaths [3]. The possible risk of pneumonia could occur as increasing usage of smoking, irregular lifestyles, chronic diseases, and weakened immune systems. Although considerable progress has been made in understanding and treatment of pneumonia over the past years [4, 5], further research and development of more effective treatment and exploring risk factors are still required to reduce the occurrence of corresponding events.

Air pollutants mainly come from automobile exhaust and industrial emission. There are many kinds of air pollutants, among which sulfur dioxide, nitrogen oxides, and total suspended particles pose the greatest threat to human health [6]. Atmospheric particulate matter (PM) can induce lung tissue damage through oxidative stress and pro-inflammatory factors [7]. Numerous epidemiological studies have been carried out and proved that atmospheric pollutants are closely related to the occurrence and death of respiratory and cardiovascular diseases [8–11]. Regarding pneumonia, the previous animal experiments have confirmed a short-term exposure to air pollutants was capable of causing lung inflammation [12]. Furthermore, recent studies demonstrated that air pollutants could increase the survival time of viral particles in the air and reduce human resistance [13], which might promote the occurrence or aggravation of pneumonia. A population study conducted in Hamilton, Canada, found a connection between long-term exposure to air pollutants and hospitalization for pneumonia in the elderly [14]. Multi-city research performed in the United States as well as found that higher concentrations of PM_10_ and ozone can enlarge the risk of pneumonia admissions [15]. In the mainland of China, a few previous investigations discovered a correlation between atmospheric pollution and hospitalization or emergency department (ED) visits for pneumonia [16–19]. To our knowledge, the previous epidemiology studies only focused on patients of specifying sources. However, there possibly have some differences in the severity of the illness among emergency patients, hospitalized patients, and outpatients; thereby, it is significant to conduct a comprehensive analysis for patients with pneumonia from multiple sources. Additionally, due to the heterogeneity of air pollution health effects in different regions [20], studies in other areas may not be directly applicable to cities like Qingdao, which have a particular pattern of atmospheric pollution and climate.

Qingdao, with 9.39 million residents, is the economic center of Shandong Province and the shipping hub of Northeast Asia. As a seaside city, Qingdao has an oceanic temperate monsoon climate. With the economic development, air pollution in Qingdao has apparent characteristics of compound pollution of soot, dust, industrial waste gas, and vehicle exhaust. The number of vehicles in Qingdao has exceeded 2.8 million, which has produced an enormous amount of vehicle exhaust pollution. Moreover, the ozone monitor on NASA’s Aura satellite found that the contamination of SO_2_ and NO_2_ in the Qingdao area is severe [21]. Apart from native pollution, long-distance transmission of particulate matter has also caused severe pollution in Qingdao [22]. Even though the air quality in Qingdao is relatively good in China, critical haze days still occur frequently. Besides, there has been no research to investigate whether there is an association between hospital visits for pneumonia and atmospheric pollution in Qingdao. Therefore, it is necessary to carry out this research to inspect the relationship under truly complex conditions.

A time stratified case-crossover design was performed to explore the relationship between short-term uncovering to atmospheric pollution and hospital visits for pneumonia in Qingdao during 2014–2018. The stratification analysis was performed in different subgroups (gender, sex, age, visit types). The primary purpose of this study is to provide more evidence for the effects of air pollution on public health in different conditions and to promote the improvement of local environmental quality.

## Methods

### Study population

The de-identified data, which contains the date of hospital visits, diagnosis, age, and sex, were extracted from the database of the affiliated hospital of Qingdao University upon approval by the Ethics committee. The board determined that informed consent was unnecessary. The affiliated hospital of Qingdao University is the largest general hospital in Qingdao, with 5.273 million annual consultations. Patients with a diagnosis of pneumonia (International Classification of Diseases, tenth revision: J12-J16, J18, J67.9, J69) were included in this study. The exclusion criteria were as follows: 1) Patients who were not verified by the results of Computed Tomography were excluded; 2) Patients with aspiration pneumonia (J69), hypostatic pneumonia (J18.201), and allergic pneumonia (J67.9) were excluded from this study; 3) To avoid hospital-acquired pneumonia caused by long-term hospitalization, we removed patients with more than two days from admission to a diagnosis of pneumonia; 4) Subsequent episode within one year of each pneumonia patient was excluded from the inquiry [23].

### Air pollutants and meteorological data

**Fig. 1:**
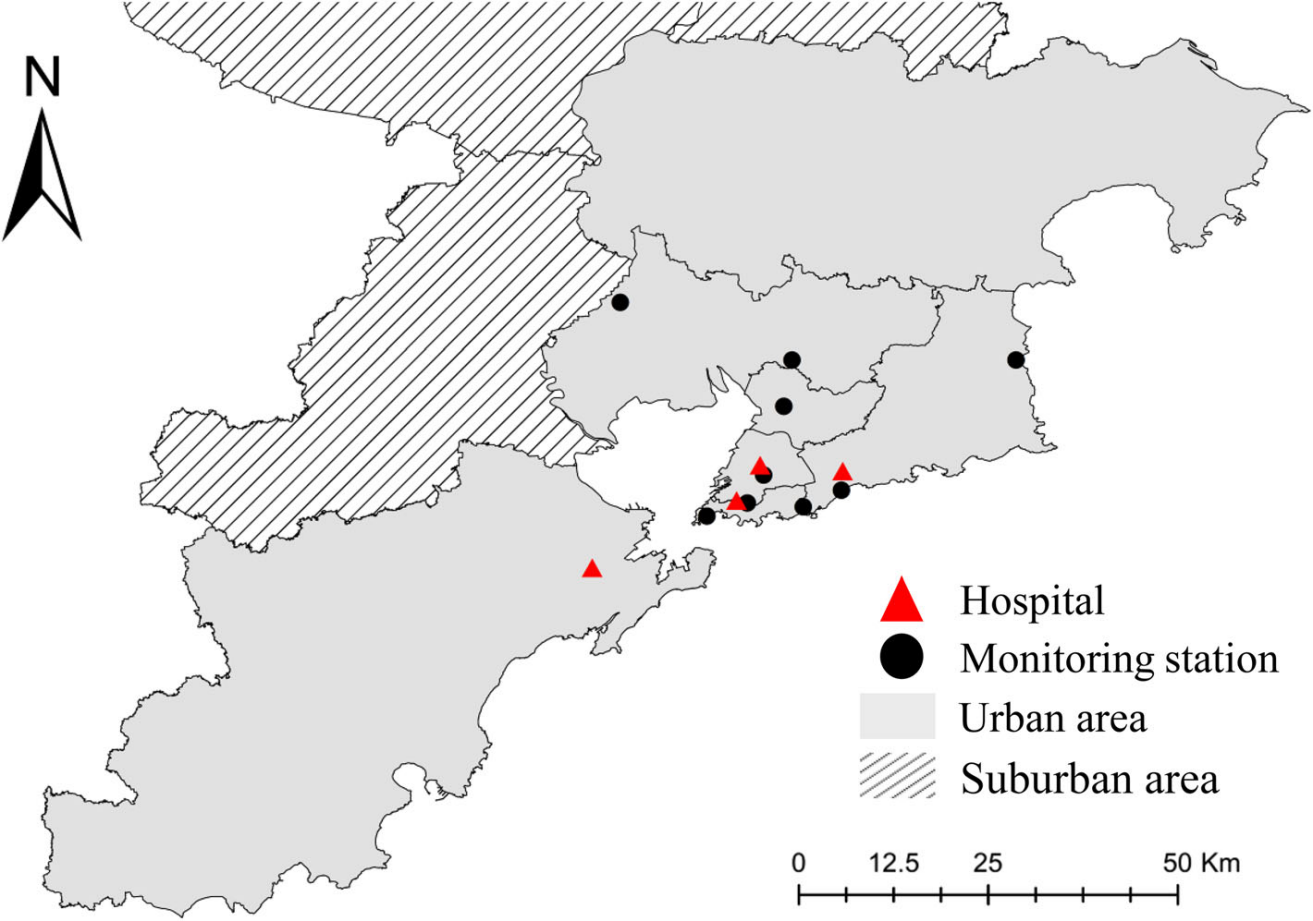
The geographical location of air quality monitoring stations and hospitals in Qingdao

Daily concentrations of atmospheric pollutants from 2014 to 2018 were obtained from the China Air Quality Monitoring and Analysis Platform. The air pollutant data were collected from nine national air quality monitoring stations. The locations of hospitals and air quality monitoring stations were marked on the map by using the software ArcGIS10.7. There are nine air quality monitoring stations in five urban districts of Qingdao city (Fig. 1). Other local monitoring stations were not used due to insufficient data credibility. The daily meteorological data were acquired from Shandong Meteorological Bureau, which includes the daily average temperature, relative humidity, and atmospheric pressure.

### Statistical analysis

The case-crossover study is an epidemiological method that was used to examine the effects of short-term exposure on the occurrence of an acute event [24]. It can be deemed as a case-control study, every patient act as their own control. Due to the self-control method, case-crossover studies can well balance the effects of many individual factors on the outcome (such as age, gender, genetic factors, etc.).

The time-stratified case-crossover design was used in this study to investigate the relationship between the acute effect of air pollutants and pneumonia occurs. The date of patients who came to the hospital was considered as the case day. The control days were defined as the same day of the week in the same month and same year as the case day, which can effectively control the day of the week (DOW), long-term trend and seasonality. On top of that, three or four control days were selected for each case. Spearman rank correlation analysis was performed to evaluate the correlation between atmospheric pollutions and meteorological factors. Conditional logistic regression models were used to estimate the odds ratio (OR) and 95% confidence interval (CI) of the correlation between atmospheric pollutants and hospital visits for pneumonia. Meteorological factors such as daily temperature, relative humidity, and atmospheric pressure were adjusted as the covariates in all models. The hysteresis effect of atmospheric pollutants has been confirmed by previous studies [25]. So, we chose the day of hospital visits (lag0) and subsequent six days (lag1-lag6) to assess the effect of air pollutants. Besides, the principal component analysis (PCA) was used to solve the multicollinearity among atmospheric pollutants. Principal components generated by PCA were added into the conditional logistic regression model to calculate the OR and 95%CI of the diverse principal components.

Stratified analysis based on sex, age, season, and visit types was applied to assess the acute effect of atmospheric pollutants on different subgroups. And the date with the most considerable odds ratio value in the single-pollutant model was chosen as the lag date of the stratification. The significant difference between different subgroups was examined by computing the 95% confidence interval on the following formula [26]:

$$ \left({E}_1-{E}_2\right)\pm 1.96\sqrt{{\left({SE}_1\right)}^2+{\left({SE}_2\right)}^2} $$

where *E*_1_ and *E*_2_ were the estimates between different subgroups, and *SE*_1_ and *SE*_2_ were their relevant standard errors.

According to Qingdao’s climatic characteristics, October to March was defined as a cold period and April to September as a warm period. The effects of atmospheric pollutants on health were expressed in terms of OR values and 95% confidence intervals. Simultaneously, the OR value was calculated based on IQR increments in each pollutant. *P* < 0.05 was recognized as the standard for statistical significance. All statistical analyses were completed on the SAS 9.4 software.

## Results

### Description of basic data

Table 1 displays the basic information of patients with pneumonia from Jan 1st, 2014 to Dec 31st, 2018. There were 4383 hospital visits with pneumonia (2313 inpatients and 2070 outpatients). Totality of these patients, approximately 56.81% were consists of the male, and 43.19% were formed of the female; 47.32% were younger than 4 years, 20.74% were aged 4 to 13 years, 20.99% were aged 14 to 59 years, 10.95% were 60 or older; 58.91% went to the hospital in the cold period, 41.09% went to the hospital in the warm period.

**Table 1:** The distribution of patients with pneumonia according to the characteristic (*n* = 4383)

Characteristics	Number of patients	Percentage (%)
Gender		
Male	2490	56.81
Female	1893	43.19
Season		
Warm	1801	41.09
Cold	2582	58.91
Age of admissions		
0–3	2074	47.32
4–13	909	20.74
14–59	920	20.99
60+	480	10.95
Visit types		
Outpatients	2070	47.23
Inpatients	2313	52.77

Warm: April to September; Cold: October to March; Outpatients: general outpatient and emergency department

Table 2 describes the basic details of atmospheric pollutants and meteorological factors from 2014 to 2018. The annual mean concentration of PM_2.5_, PM_10_, SO_2_, NO_2_, CO were 44.817 μg/m^3^, 89.466 μg/m^3^, 21.307 μg/m^3^, 35.700 μg/m^3^, 0.811 mg/m^3^. The annual concentration of PM_10_ is twice that of PM_2.5_. The annual density of PM_2.5_ and PM_10_ both surpassed the standards set by the World Health Organization (10 μg/m^3^).

**Table 2:** Summarized statistics for meteorology and air pollution in Qingdao, Shan Dong, 2014–2018

Variables	Mean	SD	Min	P_25_	Median	P_75_	Max	IQR
Air pollutants								
PM_2.5_(μg/m^3^)	44.817	33.955	4	22	35	57	304	35
PM_10_(μg/m^3^)	89.466	52.956	17	53	76	111	455	58
SO_2_ (μg/m^3^)	21.307	16.191	2	10	17	26	132	16
NO_2_ (μg/m^3^)	35.700	16.975	3	23	33	45	111	22
CO (mg/m^3^)	0.811	0.523	0.2	0.5	0.7	1	12.6	0.5
Meteorological factor								
Temperature (°C)	13.857	9.246	−11.5	5.6	14.6	22.1	30.6	16.5
Pressure (hpa)	10,081.610	89.996	9878	10,004	10,082	10,154	10,323	150
Humidity (%)	69.106	16.479	16	57	71	83	100	26

P25: lower quartile; P75: upper quartile; IQR: interquartile range

Table 3 illustrates the correlation between atmospheric pollutants and meteorological factors through the Spearman rank correlation coefficient. There was a strong positive link between PM_2.5_ and PM_10_ (R = 0.922, *P* < 0.001). Besides, a positive correlation (*P* < 0.001) was shared between air pollutants. Atmospheric particulate matter and gaseous pollutants have a negative correlation with temperature and relative humidity.

**Table 3:** Spearman correlation coefficients between air pollutants and meteorological factor during the 5-year study periods

	PM_2.5_	PM_10_	SO_2_	NO_2_	CO	Temp	RH	AP
PM_2.5_	1	0.922	0.664	0.642	0.849	−0.396	−0.132	0.284
PM_10_		1	0.66	0.686	0.795	−0.345	− 0.312	0.271
SO_2_			1	0.588	0.744	−0.508	−0.406	0.436
NO_2_				1	0.706	−0.461	− 0.493	0.469
CO					1	−0.49	− 0.237	0.419
Temp						1	0.414	−0.839
RH							1	−0.53
AP								1

All correlation coefficients were statistically significant

Temp: temperature; RH: relative humidity; AP: atmospheric pressure

### The correlation between air pollutants and pneumonia occurs

**Fig. 2:**
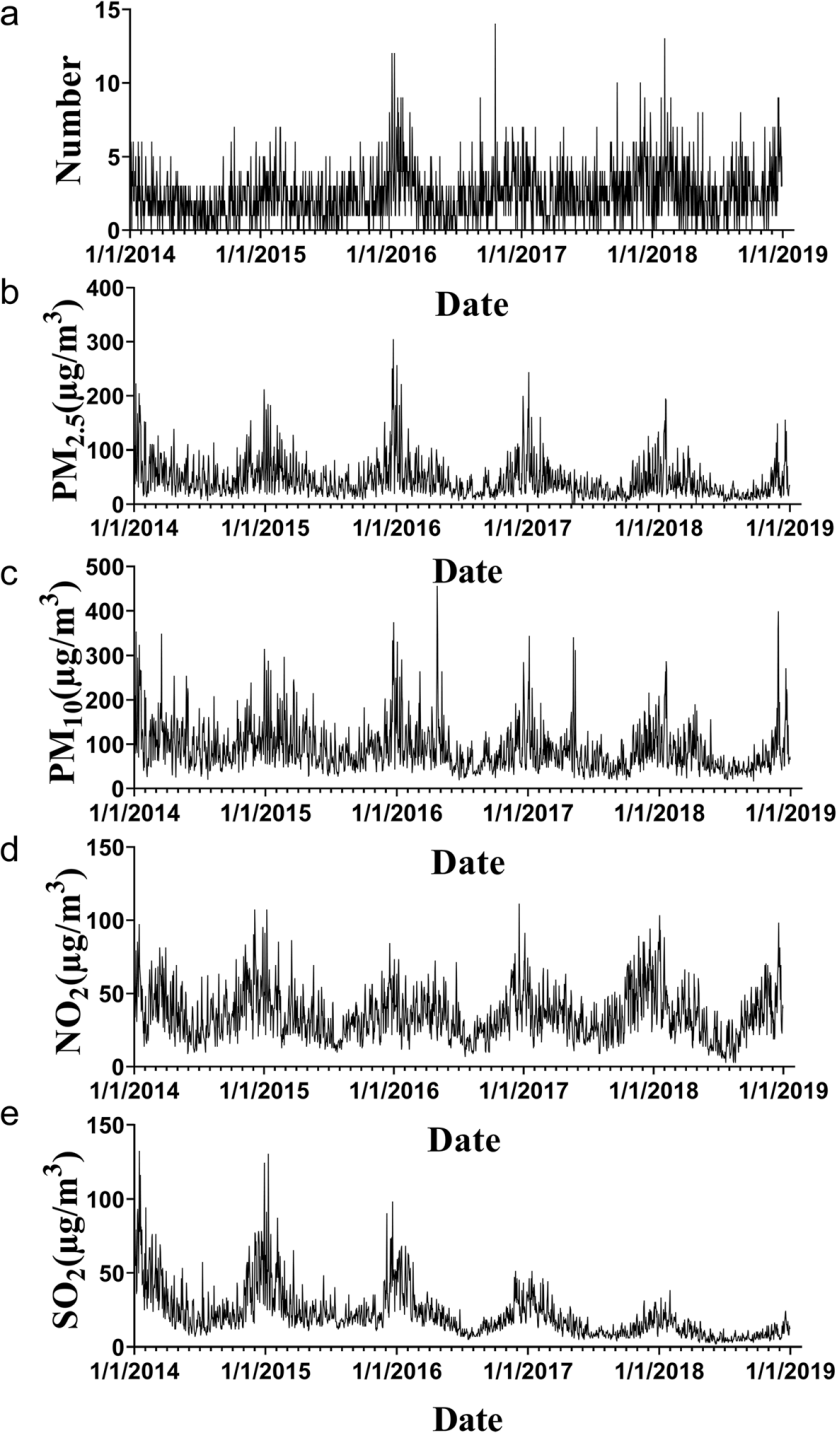
Time-series plot of the concentration of air pollutants and the number of pneumonia hospital visits during the study. Notes: (**a**) the daily number of hospital visits for pneumonia in Qingdao; (**b**) the daily concentration of PM_2.5_; (**c**) the daily concentration of PM_10_; (**d**) the daily concentration of NO_2_; (**e**) the daily concentration of SO_2_

Figure 2 shows the time series of the daily number of patients in treatment for pneumonia and the daily average concentration of atmospheric pollutants. As can be seen from the picture, there was a connection between the number of patients and air pollutants. Meanwhile, the daily visits for pneumonia and atmospheric pollution were mainly concentrated in the cold season. The concentration of SO_2_ decreased obviously over time. There was an apparent connection between the increase of atmospheric pollution concentration and hospital visits for pneumonia.

**Fig. 3:**
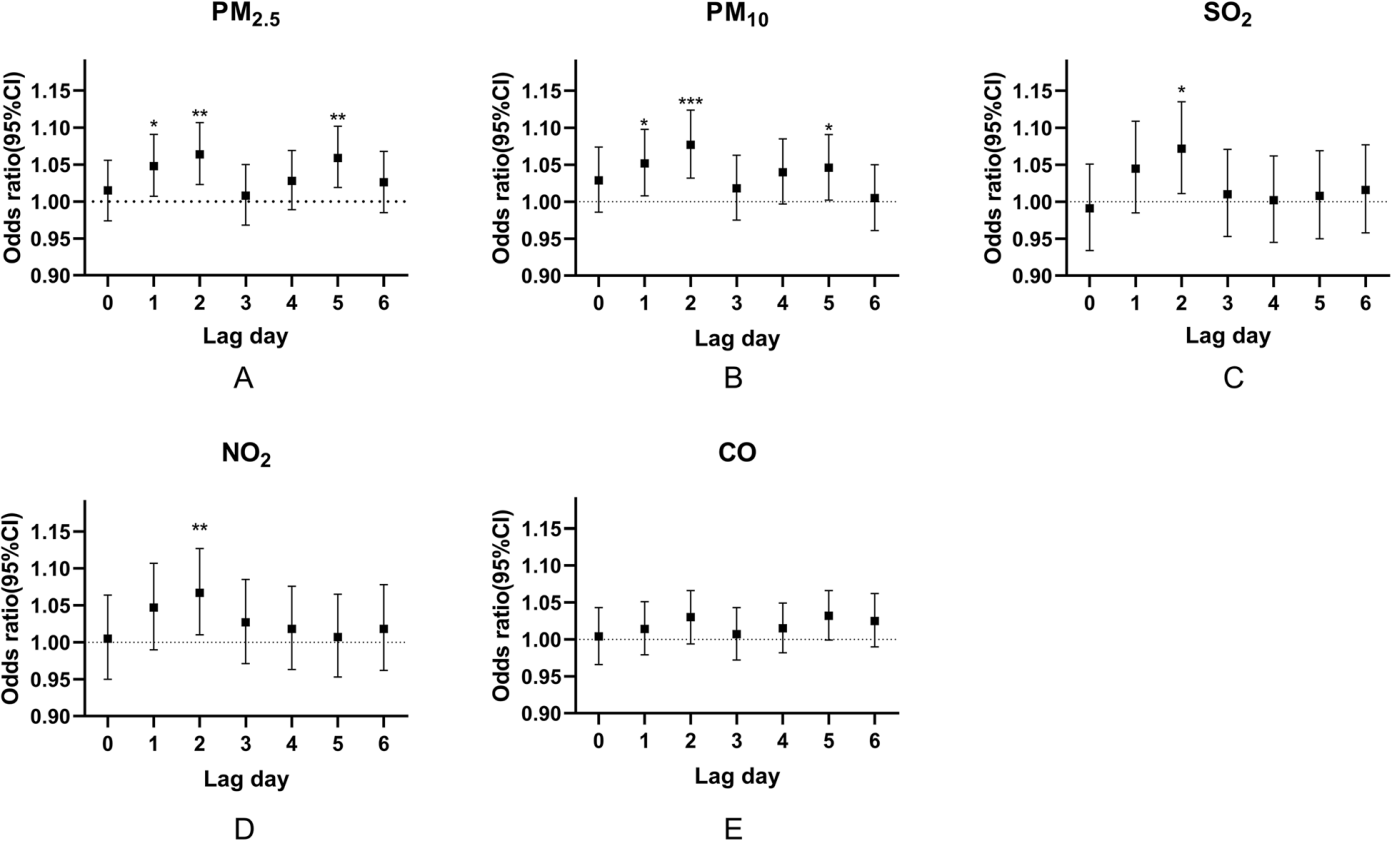
Odds ratio and 95% confidence interval for pneumonia admission associated with an interquartile range increment in air pollutant levels: Single-pollutant models. Notes: * *P* < 0.05; ** *P* < 0.01; *** *P* < 0.001. (A) PM_2.5_; (B) PM_10_; (C) SO_2_; (D) NO_2_; (E)CO

Table 4 and Fig. 3 elucidates the odds ratio and 95% confidence interval for pneumonia occurs associated with interquartile range increments in single air pollutant levels. All models were adjusted for temperature, relative humidity, and air pressure. The maximum OR values of PM_2.5_, PM_10_, SO_2_, NO_2_ were all present at the two days (lag2) before the date of hospital visits for pneumonia. When the density of PM_2.5_, PM_10_, NO_2_, and SO_2_ increased by an interquartile range at the lag2 days, the odds ratio of hospital visits for pneumonia increased by 6.4% (95%CI, 2.3–10.7%), 7.7% (95%CI, 3.2–12.4%), 6.7% (95%CI, 1.0–12.7%), and 7.2% (95%CI, 1.1–13.5%), respectively. The effect of PM_2.5_ and PM_10_ have the same pattern which peaked twice, but the other pollutants have only one peak mode.

**Table 4:** Odds ratios (with 95% CI) of hospital visits for pneumonia associated with atmospheric pollutants

Air pollutants	OR (95%CI)						
	lag0	lag1	lag2	lag3	lag4	lag5	lag6
PM_2.5_	1.015	1.048^*^	1.064^**^	1.008	1.028	1.059^**^	1.026
	(0.974,1.056)	(1.007,1.091)	(1.023,1.107)	(0.968,1.050)	(0.989,1.069)	(1.019,1.102)	(0.985,1.068)
PM_10_	1.029	1.052^*^	1.077^***^	1.018	1.04	1.046^*^	1.005
	(0.986,1.074)	(1.008,1.098)	(1.032,1.124)	(0.975,1.063)	(0.997,1.085)	(1.002,1.091)	(0.961,1.050)
SO_2_	0.991	1.045	1.072^*^	1.01	1.002	1.008	1.016
	(0.934,1.051)	(0.985,1.109)	(1.011,1.135)	(0.953,1.071)	(0.945,1.062)	(0.950,1.069)	(0.958,1.077)
NO_2_	1.005	1.047	1.067^**^	1.027	1.018	1.007	1.018
	(0.950,1.064)	(0.990,1.107)	(1.010,1.127)	(0.971,1.085)	(0.963,1.076)	(0.953,1.065)	(0.962,1.078)
CO	1.004	1.014	1.03	1.007	1.015	1.032	1.025
	(0.966,1.043)	(0.979,1.051)	(0.994,1.066)	(0.972,1.043)	(0.982,1.049)	(0.999,1.066)	(0.990,1.062)

Note: All conditional logistic models were adjusted for temperature, relative humidity, and atmospheric pressure. The concentration of each pollutant increases in interquartile intervals

Lag represents the days before the hospital visits. lag0 represents the day of the hospital visits; Lag1 represents one day before hospital visits; the others so on

^*^P < 0.05; ^**^ *P* < 0.01; ^***^ *P* < 0.001

### Stratification analysis

Table 5 summarizes the association between pneumonia hospital visits and air pollutants divided by gender, age, season, and types of the visitation. Both PM_2.5_ and PM_10_ had a noticeable influence on the attack of pneumonia for male and female, but the difference between them was not significant. The impact of atmospheric particulates on different ages mainly occurs in patients younger than 4 years old. For children aged 0 to 3, an IQR increment of PM_10_ was associated with a 10.1% (95%CI, 3.7–16.9%) increase in the odds ratio of hospital visits for pneumonia. For the season, the OR value of each pollutant in the cold season was higher than that in the warm season, and NO_2_ has a statistical difference between cold and warm periods. An IQR increment of NO_2_ in the cold season was related to a 9.8% (95%CI, 3.3–16.8%) rise in the odds ratio of patients who came to the hospital. Regarding the way of visiting, the OR value of outpatients increased more significantly with the IQR increase of atmospheric pollutant concentration than that of inpatients.

**Table 5:** Association between atmospheric pollutants and patients for pneumonia in different subgroups

Subgroup	OR (95%CI)			
	PM_2.5_	PM_10_	NO_2_	SO_2_
Gender				
Male	1.061 (1.006,1.119) ^*^	1.077 (1.017,1.140) ^*^	1.085 (1.007,1.168) ^*^	1.051 (0.972,1.136)
Female	1.069 (1.008,1.134) ^*^	1.077 (1.011,1.147) ^*^	1.046 (0.962,1.136)	1.098 (1.007,1.197) ^*^
Age				
0–3	1.075 (1.017,1.136) ^*^	1.101 (1.037,1.169) ^a **^	1.096 (1.012,1.187) ^*^	1.066 (0.986,1.153)
4–13	1.025 (0.939,1.119)	0.984 (0.893,1.085)	1.035 (0.916,1.170)	1.050 (0.916,1.205)
14–59	1.087 (0.991,1.191)	1.104 (1.002,1.217) ^*^	1.062 (0.942,1.198)	1.120 (0.973,1.288)
60+	1.072 (0.945,1.217)	1.121 (0.989,1.271)	1.029 (0.870,1.217)	1.077 (0.902,1.285)
Season				
Warm	0.985 (0.871,1.115)	1.001 (0.909,1.102)	0.946 (0.830,1.078)	0.985 (0.803,1.207)
Cold	1.072 (1.028,1.118) ^**^	1.095 (1.043,1.149) ^***^	1.098 (1.033,1.168) ^b **^	1.086 (1.022,1.153) ^**^
Visit types				
outpatients	1.080 (1.020,1.143) ^**^	1.092 (1.028,1.161) ^**^	1.063 (0.982,1.150)	1.074 (0.984,1.173)
inpatients	1.050 (0.994,1.109)	1.063 (1.002,1.127) ^*^	1.070 (0.991,1.156)	1.069 (0.990,1.154)

Note: All conditional logistic models were adjusted for temperature, relative humidity, and air pressure. The concentration of each pollutant increases in interquartile intervals

^a^Effects of atmospheric pollutants significantly differ in 0–3 and 4-13 years old children; ^b^ Effects of atmospheric pollutants significantly differ in warm seasons and cold seasons

^*^P < 0.05; ^**^ *P* < 0.01; ^***^ *P* < 0.001

### Principal component analysis

**Fig. 4:**
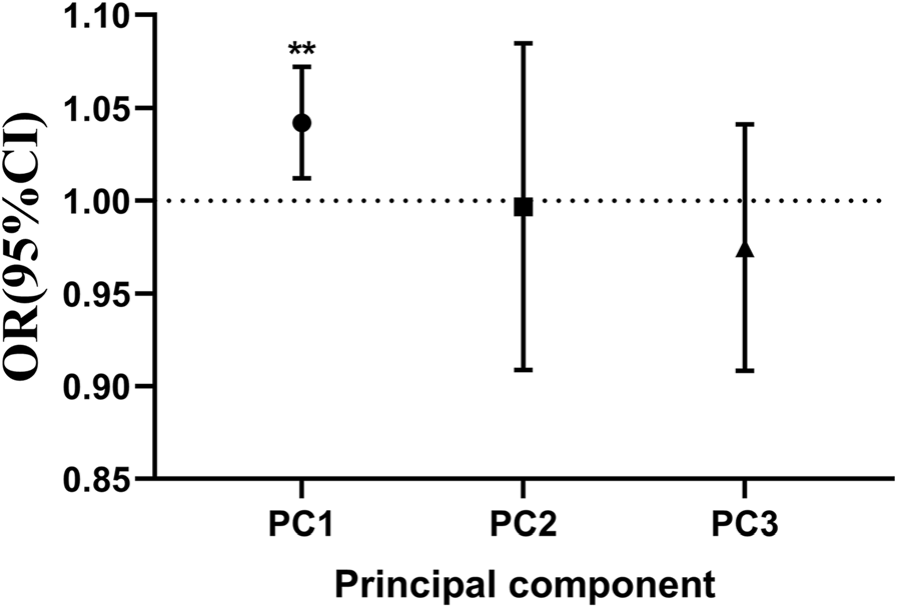
Odds ratio and 95% confidence interval of hospital visits for pneumonia induced by principal component of atmospheric pollutants. Notes: ** *P* < 0.01; PC1: the first principal component; PC2: the second principal component; PC3: the third principal component; OR: odds ratio; CI: confidence interval

Table 6 shows the eigenvalues of atmospheric pollutants on lag 2 days and relevant eigenvectors. The eigenvalue of the first principal component was 3.05 (higher than 1), offering 76.27% of pollutant information; the value of the eigenvector of diverse atmospheric pollutants was all positive values, and PMs were higher than NO_2_ and SO_2_. The second and third principal components supplied a similar proportion of the atmospheric pollutant information, but their eigenvalue was smaller than 1. Table 7 demonstrates the factor loadings of various pollutants on the principal components. To better explain the original data, the three principal components were selected as the complex index to represent the atmospheric pollutants. Figure 4 displays the odds ratio and 95% confidence interval of hospital visits for pneumonia when the principal component was included in the conditional logistic model. The odds ratio of the first principal component was 1.042 (95%CI, 1.012–1.072). The other principal components have a non-significant effect on trigger the hospital visits for pneumonia.

**Table 6:** Coherent matrix eigenvalues of multiple atmospheric pollutants model on lag2 days and relevant eigenvectors

	PC1	PC2	PC3	PC4
Eigenvalue	3.051	0.462	0.397	0.089
Proportion of variance	0.763	0.116	0.099	0.022
Cumulative proportion of variance	0.763	0.879	0.978	1.000
PM_2.5_	0.535	−0.196	−0.398	−0.719
PM_10_	0.529	−0.302	− 0.388	0.691
SO_2_	0.457	0.885	0.055	0.068
NO_2_	0.474	−0.294	0.829	−0.026

PC1: the first principal component; PC2: the second principal component; PC3: the third principal component; PC4: the fourth principal component

**Table 7:** The loading matrix of atmospheric pollutants on three principal components

Variables	PC1	PC2	PC3
PM_2.5_	0.934	−0.133	− 0.251
PM_10_	0.925	−0.205	−0.245
SO_2_	0.797	0.602	0.034
NO_2_	0.829	−0.200	0.523

PC1: the first principal component; PC2: the second principal component; PC3: the third principal component

## Discussion

The purpose of this study was to investigate the relationship between the concentration of air pollutants and the occurrence of pneumonia. The major discovery of our research was that the risk of hospital visits for pneumonia increases with an IQR increment of atmospheric pollutant levels (PM_2.5_, PM_10_, SO_2_, NO_2_). The effects of air pollutants were slightly different in diverse subgroups. The effect of atmospheric mainly arose in the cold period rather than the warm period. Younger children might be more sensitive to atmospheric pollutants. These findings could better explain the effect of air pollutants on human health.

In previous studies, the research mainly focused on the health effects of atmospheric pollution, especially PM. Many recent investigations found that the rise of the air pollutant levels had a positive relationship with the incidence and mortality of respiratory diseases, including asthma, upper respiratory infections, and pneumonia [27–30]. This research, like previous singular-source studies, discovered a connection in atmospheric pollutants and hospital visits with pneumonia. With an IQR increment of PM_2.5,_ PM_10_, NO_2_, and SO_2_ levels, the odds ratio of ED visits with pneumonia for each air pollutant increased by 14.0, 10.9, 14.1, and 4.5% in Kaohsiung City on lag three days [25]. Duan et al. [16] reported a significant relationship between increased PM_2.5_ concentrations and hospitalization for pneumonia in Shijiazhuang during 2013, and the effect of PM_2.5_ was highest on the day of admission (OR = 1.011, 95%CI: 1.005–1.017). Even though studies have found an association between atmospheric pollutants and pneumonia, the results on the hysteresis effect of pollutants were not identical. Sue et al. [31] once implied that there was a correlation between the chemical properties of air pollutants and the risk of hospitalization. The composition and sources of air pollutants in different regions were different, which might be the reason for the inconsistent lag effects of air pollution in distinct areas. Another possible explanation might be the difference in the structure and characteristics of the population.

The mechanism of pneumonia caused by air pollution is not known adequately. Animal experiments have proved that exposure to atmospheric particulates would reduce the antibacterial ability of the lungs and aggravate the original pulmonary inflammation [32]. NO_2_ also could provoke changes in pulmonary immune function by causing damage to bronchial and alveolar epithelial cells [33]. PM could reduce the activity of pulmonary macrophages and epithelial cells, and play a role in promoting inflammation as well as oxidative stress [34]. Furthermore, exposure to PM would inhibit pulmonary macrophages, which increases the susceptibility of the lungs to infection [35]. Besides, experiments had shown that exposure to atmospheric particles would cause the production of reactive oxygen species (ROS). ROS would produce a series of cellular responses (Mitochondrial damage [36], inflammatory mediators release, apoptosis), and eventually lead to the occurrence of disease [37]. Except for atmospheric particulates, SO_2_ exposure also could induce lung mitochondrial dysfunction, resulting in cellular dysfunction and lung illness [38]. These studies implied that air pollution might cause pneumonia by inducing the abnormal immune function of the lungs and producing oxidative damage.

Gender and age differences had always been the focus of environmental epidemiological research. In this study, we found that the impact of atmospheric particulate matter on men and women was both statistically significant, but the difference between them was not significant. This result was consistent with some previous studies. An epidemiological survey conducted in Shenzhen found that air pollution affected the occurrence of respiratory diseases both on men and women, but there had no significant gender difference [39]. However, there were some studies with different results. Duan et al. [16] concluded that men were more susceptible to the effect of PM_2.5_ and PM_10_ on pneumonia hospitalization, but their interaction *P* value was not estimated. Some research detected that the impact of PM_2.5_ on pneumonia hospitalization was more influential in women than men [40, 41]. In terms of age stratification, infants less than 1-year-old were proved to more sensitive to PM_2.5_ and PM_10_ in a study [17]. Cheng et al. [25] found children older than four years were more vulnerable to PM_2.5_ than those younger than four years. In this investigation, we found that children younger than four years old seem more susceptible to atmospheric pollutants. The reason why younger children have a higher odds ratio might be due to the vulnerability of airways and alveoli, immature immune systems, and high rates of respiratory infections, which finally leads to their high sensitivity to the atmospheric pollutants. Most studies had inconsistent results in terms of age and gender. The possible reason for this might be owing to the inclusion-exclusion criteria, the different composition of air pollutants, and the number of cases in various studies.

On the seasons, the concentration of air pollutants and the number of hospital visits for pneumonia both increased during the cold season. On the one hand, this was because of the increased emissions of pollutants caused by winter heating and the transmission pollutants from the northwest Shandong. On the other, Dong et al. [42] found a clear seasonal trend in the total microbial level in Qingdao, and the total microbial concentration was significantly increased with the increase of the intensity of air pollutants in winter. This phenomenon might be a crucial reason for the rise in the number of patients who were admitted to the hospital for pneumonia in winter. Consistent with the time series results, the impacts of atmospheric pollutants on the occurrence of pneumonia mainly arose in the cold period. This result was similar to prior studies that identified a more potent effect of air pollutions in winter [18, 43]. Low temperature would reduce the ability of the respiratory system to resist infection, which might relate to the decrease of cilia clearance ability of the respiratory system and leukocyte phagocytosis [44]. In consequence, people might be more likely to catch pneumonia in winter, and the effects of atmospheric pollutants were more visible. However, in other people’s research, they found that atmospheric pollutants were more noticeable during the warm or transitional season [16, 45]. The reason for this regional difference was possibly owing to seasonal changes in the composition of atmospheric pollutants, people’s lifestyles in various regions, and local meteorological conditions. Besides, as the pollution was severe in winter in the Qingdao area, the high incidence of haze in winter might aggravate the harm of air pollution to the human body in Qingdao.

To our knowledge, previous research about pneumonia only added air pollutants into multi-pollutant models without considering the impact of strong correlations between air pollutants. However, the collinearity between atmospheric pollutants might lead to incorrect estimates or unstable models [46]. Besides, the air pollutants in China mainly come from the combustion of fuel, there are apparent homologous characteristics among these pollutants, so it has little significance to use the multi-pollutant model directly. As a result, we used a principal component analysis to investigate the relationship between multiple atmospheric pollutants and hospital visits for pneumonia. In the principal component regression model, the effect of the first principal component, which stands for the mixed pollutants was significantly on inducing the hospital visits for pneumonia. This result possibly signifies that it was the mixed pollution (PM_2.5_, PM_10_, NO_2_, SO_2_) induced hospital visits for pneumonia, and atmospheric particulate matter was the most important factor of them. Previous research proved that the mixed effect was mainly driven by PM [47]; perhaps this was the reason why PM was more vital in inducing hospital visits for pneumonia. Besides, the former epidemiology research found that PM_2.5_ was more toxic than PM_10_ [48], which possibly the reason why the effect of PM_2.5_ was higher than PM_10_.

The strength of this research was that it covers a wide range of people while preceding studies in China have mainly concentrated on specific source crowds. Additionally, the principal component analysis could better explain the effect of various atmospheric pollutants in causing pneumonia. There also have some shortcomings in this study: Foremost, due to the confidentiality of the data, information including the patient’s home address, indoor air pollutants and behavior habits (such as smoking, drinking) cannot be obtained. Thus, it was impossible to assess individual exposure accurately. Second, the data of hospital visits for pneumonia was only obtained from four districts of one hospital, thus it only partially accounted for the total patients in the city. Although it does not affect the judgment of the relationship between air pollutants and hospital visits for pneumonia, the lack of complete case data may reduce the accuracy of the estimation of the results. At last, owing to the heterogeneity of various cities, the result of a single town was difficult to apply to other areas. Therefore, more experiments were needed in the future to verify the mechanism of air pollution on pneumonia occurs.

## Conclusions

In short, this study found a significant correlation between short-term uncovering to PM_2.5_, PM_10_, NO_2_, SO_2_ and hospital visits for pneumonia in Qingdao. The effect of atmospheric pollutants mainly arose in a cold period. The particulate matter might the principal reason in inducing hospital visits for pneumonia.

## Data Availability

The pollutant data are available from the China Air Quality Monitoring and Analysis Platform (www.aqistudy.cn). The Meteorological data are available from the Shandong Meteorological Bureau (http://sd.cma.gov.cn/). The data of pneumonia patients are not publicly available due to the requirement of the affiliated hospital of Qingdao University but are obtainable from the corresponding author upon legitimate request.

## Abbreviations

PM: Particular matter
PM_2.5_: Fine particulate matter with aerodynamic diameter less than 2.5μm
PM_10_: Particulate matter with aerodynamic diameter less than 10μm
SO_2_: Sulfur dioxide
NO_2_: Nitrogen dioxide
CO: Carbon monoxide
ROS: Reactive oxygen species
ED: Emergency department
OR: Odds ratio
CI: Confidence interval
IQR: Interquartile range
PCA: Principal component analysis
